# Aspects Concerning Parallel Robots Used in Rehabilitation

**DOI:** 10.3390/bioengineering12111224

**Published:** 2025-11-09

**Authors:** Adrian Todor, Daniel Vasile Banyai, Cornel Brisan, Adriana Daniela Banyai

**Affiliations:** 1 Department of Orthopaedics and Traumatology, “Iuliu Hațieganu” University of Medicine and Pharmacy—Cluj-Napoca, 400012 Cluj-Napoca, Romania; adrian.todor@umfcluj.ro; 2 Department of Mechanical Engineering, Technical University of Cluj-Napoca, 400114 Cluj-Napoca, Romania; 3 Department of Mechatronics and Machine Dynamics, Technical University of Cluj-Napoca, 400114 Cluj-Napoca, Romania; cornel.brisan@mdm.utcluj.ro (C.B.); adriana.tomsa@campus.utcluj.ro (A.D.B.)

**Keywords:** parallel mechanisms, rehabilitation robotics, AHP, simulation

## Abstract

This study presents a comprehensive simulation-based comparative analysis of four parallel robotic mechanisms, each developed to assist patient recovery through adaptive movement control and feedback, particularly for upper and lower limb therapy. Kinematic and dynamic models were developed and implemented in Matlab-Simulink, integrating force control via conventional regulators and real-time interaction with simulated patient-applied forces. The structural differences between spherical, rotational, and universal joints in each kinematic chain variant were evaluated. To systematically determine the most suitable design, a detailed Analytic Hierarchy Process was applied considering performance, precision, stability, and actuator effort. The study emphasizes the advantages of parallel robots in rehabilitation due to their precision, rigidity, and compact design, highlighting the potential of parallel robotic systems in customized and adaptive physical therapy interventions. These insights contribute to the optimal design selection of clinical motor therapy robots.

## 1. Introduction

The increasing global prevalence of motor disabilities has accentuated the need for efficient, adaptable, and scalable rehabilitation solutions. According to the World Health Organization, approximately 15% of the global population lives with some form of disability, and between 110 and 190 million individuals experience significant limitations in movement [1]. These impairments, frequently resulting from strokes, traumatic injuries, or neurological disorders, can severely affect independence, mobility, and quality of life.

In response to this societal and medical challenge, robotic rehabilitation has emerged as a transformative technological field. Robotic systems offer consistent, repetitive, and quantifiable movement therapy, which has been shown to support neural reorganization and improve motor recovery outcomes [2,3]. A wide range of devices, from wearable exoskeletons to stationary end-effector-based platforms, have been developed to target specific motor functions of the upper and lower limbs [4,5].

Our prior systematic review [6] explored the landscape of robotic systems in rehabilitation, revealing several key findings:

- Robotic therapies are clinically effective in improving strength, coordination, and sensorimotor function, especially when applied intensively and repetitively;
- Integration with feedback systems (haptic, visual, auditory) enhances patient engagement and facilitates neuroplasticity;
- Challenges include high acquisition costs, the need for therapist training, and limited device personalization.

While prior studies have thoroughly documented robotic device categories and their application scopes, there is limited focus on the mechanical design trade-offs between different kinematic architectures, particularly parallel mechanisms, which offer unique advantages in terms of rigidity, precision, and dynamic performance [7,8,9,10].

Unlike serial robotic arms, which suffer from cumulative errors and reduced rigidity, parallel robotic mechanisms connect a fixed base to a mobile platform via multiple independent kinematic chains operating simultaneously, each contributing to the overall degrees of freedom [11,12,13]. This configuration results in the following:

- High structural stiffness and reduced sensitivity to external disturbances;
- Improved dynamic responsiveness and motion repeatability;
- Compact design, enabling smaller devices with wider workspaces [14,15].

These attributes are particularly relevant in rehabilitation contexts, where safety, control precision, and adaptive interaction are essential for assisting or resisting joint movement during therapy sessions [16,17,18].

Despite their theoretical and practical potential, few studies have systematically compared different parallel kinematic configurations under rehabilitation-specific operating conditions. Several systematic reviews and experimental investigations from the recent literature support the use of parallel mechanisms in rehabilitative contexts. Dong et al. [19] conducted a comprehensive review of parallel ankle rehabilitation robots, highlighting their ability to deliver uniform training of the ankle joint complex (AJC) while maintaining control over multi-directional motion and applied forces. More specialized designs, such as soft parallel robots actuated by pneumatic artificial muscles, have been shown to improve safety in human–robot interactions while preserving mechanical compliance [20].

Various kinematic configurations have been proposed to optimize the mechanical and functional properties of such robots [21]. For example, Li et al. [22] introduced a novel parallel robot for ankle rehabilitation and performed Jacobian-based optimization to enhance its usable workspace and avoid kinematic singularities. In another study, Du et al. [23] highlighted how spherical parallel mechanisms can better replicate natural joint kinematics, making them highly suitable for multiaxial joints such as the wrist and ankle.

One of the consistent challenges in this domain lies in selecting the most appropriate kinematic configuration, each offering trade-offs regarding force distribution, control complexity, structural compactness, and spatial coverage. Redundantly actuated or decoupled mechanisms, as discussed in [18,20], have proven effective in increasing operational safety and system fault tolerance, but at the cost of increased design complexity and energy consumption. Moreover, the selection of a suitable configuration often lacks a quantitative multicriterial evaluation framework to account for conflicting objectives such as motion fidelity vs. mechanical simplicity [24,25,26].

To overcome this limitation, the present study proposes a simulation-driven and a multi-criteria analysis of four distinct parallel robotic architectures, each combining with translational actuators (T) and different sequences of spherical (S), rotational (R), and cardan/universal (C) joints: STR, STC, TSR, and TSC. These configurations differ in how they distribute motion and force across the kinematic chain, directly impacting rehabilitation-relevant factors, such as the following:

- Motion tracking precision and stability;
- Actuator force requirements;
- Velocities that actuators have to deliver; together with the required forces, they determine the energy produced by the actuators.

To evaluate these factors objectively, we implemented each 3D model in Matlab-Simulink (Matlab Version 23.2; Release 2023b; License Academic) and simulated their behavior under the same dynamic conditions, including motion commands and external perturbations.

The Analytic Hierarchy Process (AHP), a robust decision-making methodology, was used to integrate and rank the simulation results. AHP enables a weighted, criteria-based comparison by combining both objective simulation metrics (e.g., tracking error, actuator effort) and qualitative engineering judgments (e.g., mechanical complexity, feasibility) [24,25,26].

While structural rigidity, motion precision, and control responsiveness are well-documented advantages of parallel mechanisms, their real-world clinical integration also depends on practical constraints such as energy consumption and power source flexibility. In mobile or modular rehabilitation settings, such as home-based therapy or overground gait training, battery-powered robotic systems offer significant logistical and ergonomic advantages. These include untethered operation, reduced infrastructure dependence, and improved patient comfort [27]. Designing for battery feasibility also influences joint selection and kinematic topology. For instance, configurations with lower joint friction (e.g., spherical vs. rotational) and reduced angular travel minimize unnecessary energy losses. These considerations reinforce the relevance of integrating power efficiency metrics into the multi-criteria evaluation process of robotic architectures. By prioritizing designs that minimize actuator stress and energy peaks, the path toward portable, battery-powered rehabilitation platforms becomes technically achievable and clinically viable.

This work offers a complete framework for selecting the optimal parallel mechanism for motor rehabilitation applications, balancing engineering constraints (simulation fidelity) with clinical requirements (structured decision analysis). The methodology can also be extended to support future hardware prototyping and clinical validation based on simulation-optimized design choices.

## 2. Materials and Methods

### 2.1. Mechanisms Configurations

The four proposed mechanisms have 3 degrees of freedom (DOF) constituting parallel robotic rehabilitation platforms. Each mechanism consists of three identical legs (kinematic chains) connecting the fixed base to the moving platform. Each leg integrates a specific sequence of joints: spherical (S), translational/prismatic (T), rotational (R), or cardan/universal (C), leading to different mechanical properties.

To rigorously determine the mobility (degrees of freedom) of each variant, we apply the Kutzbach–Grübler mobility criterion for spatial mechanisms:

(1) $$DOF=6\cdot \left(n-1\right)-\sum_{i=1}^{3}f_{i}\cdot \left(6-m_{i}\right),$$

where n is the number of rigid bodies (including the base), fᵢ is the number of joints of type “i”, and mᵢ is the number of DOF allowed by joint of type “i”.

To describe the motion of the mobile platform, two coordinate frames are defined: one fixed frame B (X0, Y0, Z0) attached to the base, and one mobile frame P (X, Y, Z) located at the center of mass of the moving platform. The base coordinate frame B serves as the global reference, while the platform coordinate frame P moves with the platform and is used to describe the local positions of the platform attachment points. Each leg connects point B_i_ on the fixed base to point P_i_ on the moving platform, where i = 1, 2, 3. These points define the positions of the spherical, translational joints and the cardan or revolute joints on the platform, depending on the configuration. The three platform joints, P_i_, and the three fixed base points, B_i_, are positioned symmetrically at 120° intervals around the central axis.

**Figure 1:**
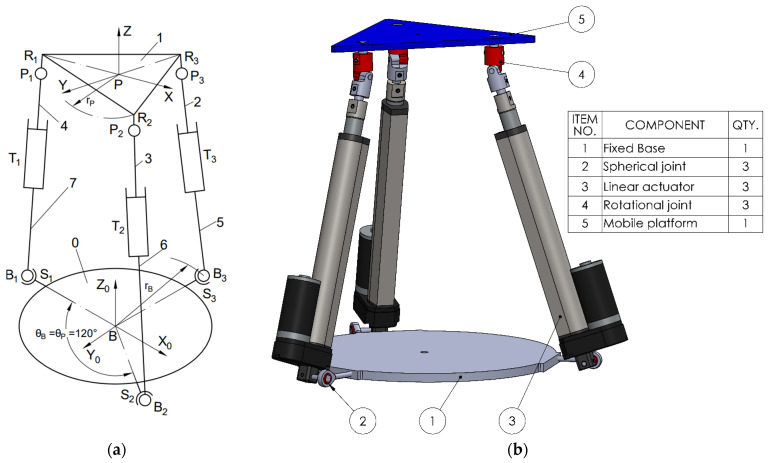
The 3STR mechanism. (**a**) Schematic; (**b**) 3D CAD model.

The first analyzed variant of the kinematic chain (leg) consists of a spherical joint (S), (3 DOF) at the base, a linear actuator (T), (1 DOF), and a rotational joint (R), (1 DOF) at the connection with the mobile platform. The parallel mechanism is composed of three such identical kinematic chains; therefore, the number of joints of each type fᵢ is 3 (3STR). The total number of rigid bodies, including the base, mobile platform, and intermediate links, is n = 8 (as illustrated in Figure 1 where the displacements made by the prismatic actuators are denoted as q_1_, q_2_, and q_3_).

**Figure 2:**
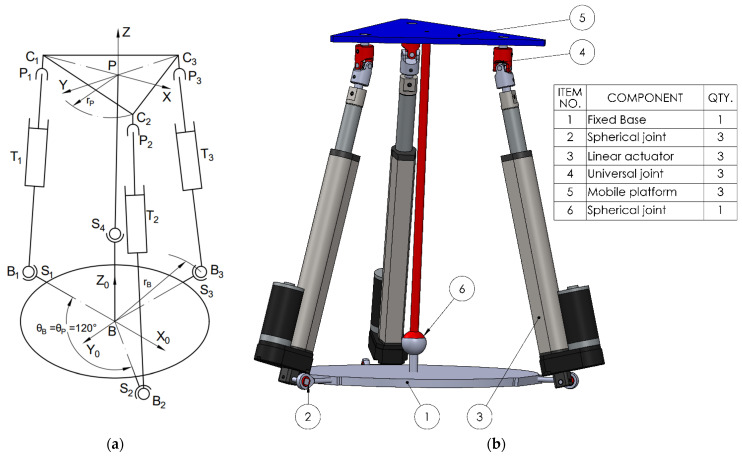
The 3STC mechanism. (**a**) Schematic; (**b**) 3D CAD model.

The second kinematic chain configuration consists of a spherical joint (S), (3 DOF) at the base, a linear actuator (T), (1 DOF) as the active element, and a cardan (universal) joint (C), (2 DOF) that connects to the mobile platform (see Figure 2), (STC). Additionally, a central spherical joint (3 DOF) is introduced between the mobile platform and the fixed base, aligned with the centers of mass. This additional spherical coupling acts as a passive constraint that improves stability and alignment. The total number of joints becomes 10, accounting for the central coupling components.

**Figure 3:**
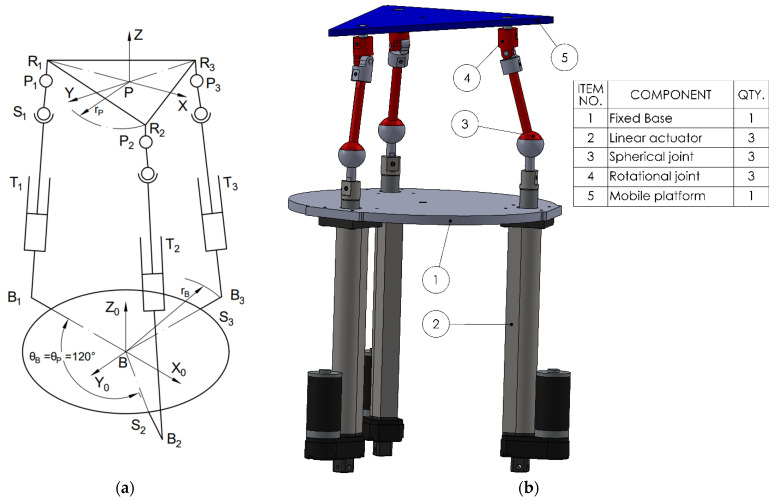
The 3TSR mechanism. (**a**) Schematic; (**b**) 3D CAD model.

**Figure 4:**
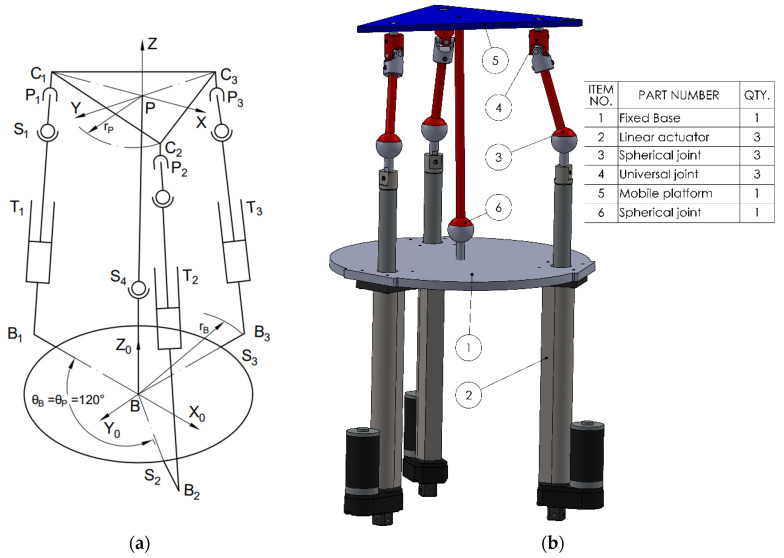
The 3TSC mechanism. (**a**) Schematic; (**b**) 3D CAD model.

The third variant (3TSR) consists of a linear prismatic actuator (T), directly connected at the base, followed by a spherical joint (S) in the middle of the leg, and a rotational joint (R) at the platform interface (see Figure 3).The fourth configuration (see Figure 4) (TSC) consists of a translational joint at the base (T), (1 DOF), a spherical joint (S), (3 DOF), and a cardan joint (C), (2 DOF) that connects to the mobile platform. As in the STC variant, a central spherical joint is also added between the mobile platform and the base. Applying the mobility criterion yields a result of 3 degrees of freedom, allowing two rotational motions and one translational displacement, for all the four configurations.

### 2.2. Inverse Kinematics

The inverse kinematics problem for the proposed 3 DOF rehabilitation platform consists in determining the required linear displacements of the three actuated prismatic joints (q_1_, q_2_, q_3_), so that the mobile platform reaches a specified position and orientation P within the task space [28].

The position of the i-th attachment point P_i_ on the moving platform is defined as follows:

(2) $$\vec{P_{i}}=\left[\begin{array}{c} r_{P}\;\cos\;\left(\frac{2\pi }{3}\left(i-1\right)\right)\\ r_{P}\;\sin\;\left(\frac{2\pi }{3}\left(i-1\right)\right)\\ 0 \end{array}\right],\;for\;i=1,\;2,\;3,$$

The corresponding base joint positions, B_i_, in the base coordinate frame are given by the following:

(3) $$\vec{B_{i}}=\left[\begin{array}{c} r_{B}\;\cos\;\left(\frac{2\pi }{3}\left(i-1\right)\right)\\ r_{B}\;\sin\;\left(\frac{2\pi }{3}\left(i-1\right)\right)\\ 0 \end{array}\right],\;for\;i=1,\;2,\;3,$$

The complete pose of the moving platform in the base frame is described by the position vector, r_0_, which represents the Cartesian coordinates of the origin of the mobile platform in the base frame, so that horizontal translations are null, hence

(4) $$\vec{d}=\left[\begin{array}{c} 0\\ 0\\ z \end{array}\right],$$

and the rotation matrix *R* from the platform frame to the base frame, which governs the transformation of vectors defined in the local frame of the platform to the global base coordinate frame, is as follows:

$$R=R_{y}\left(\theta \right)R_{x}\left(\varphi \right),$$

(5) $$R=\left[\begin{array}{ccc} \cos\left(\theta \right) & \sin\left(\varphi \right)\sin\left(\theta \right) & \cos\left(\varphi \right)\sin\left(\theta \right)\\ 0 & \cos\left(\theta \right) & -\sin\left(\theta \right)\\ -\sin\left(\theta \right) & \sin\left(\varphi \right)\cos\left(\theta \right) & \cos\left(\varphi \right)\cos\left(\theta \right) \end{array}\right]$$

The i-th leg vector $\vec{L_{i}}$, which represents the length of each leg is computed as follows:

(6) $$\vec{L_{i}}=d+R\cdot \vec{P_{i}}-\vec{B_{i}}\;,\;for\;i=1,\;2,\;3,$$

Given the desired position and orientation of the platform, the required actuator length/displacement q_i_ can be computed for each leg as follows:

(7) $$q_{i}^{2}={\left(B_{xi}+P_{xi}\cdot \cos\left(\theta \right)+P_{yi}\cdot \cos\left(\varphi \right)\sin\left(\theta \right)\right)}^{2}+{\left(B_{yi}+P_{yi}\cdot \cos\left(\varphi \right)\right)}^{2}+\;{\left(z+B_{zyi}\cdot \sin\left(\theta \right)+P_{zi}\sin\left(\varphi \right)\cos\left(\theta \right)\right)}^{2}\;or\;q_{i}=\left\|L_{i}\right\|$$

### 2.3. Jacobian Matrix

The Jacobian matrix establishes the relationship between the linear velocities of the prismatic actuators ($\dot{q_{1}}$, $\dot{q_{2}}$, $\dot{q_{3}}$) and the generalized velocity of the moving platform.

In parallel robots with decoupled translational and rotational DOF, such as the configurations studied in this work, the Jacobian can be formulated to map the platform’s motion to actuator displacements as follows [29]:

(8) $$\dot{q}=J\cdot {\dot{X}}_{p},$$

where

$\dot{q}={\left[\dot{q_{1}},\;\dot{q_{2}},\dot{q_{3}}\right]}^{T}$ are the velocities of the linear actuators;
${\dot{X}}_{p}={\left[\dot{z},\;\omega_{x},\;\omega_{y}\right]}^{T}$ are the two angular velocities and the linear velocity of the mobile platform.

Equation (8) can be rewritten to express the actuator velocities $\dot{q}$ as a function of the platform’s generalized velocity, ${\dot{X}}_{p-0}$ as follows:

(9) $$\dot{q}=J_{A}\cdot {\dot{X}}_{p-0}=J_{IA}\cdot {\vec{V}}_{Tj},$$

Here, *J_A_* is the analytical Jacobian matrix that links the platform to the actuated joint velocities.

(10) $${\vec{V}}_{Tj}=J_{IIA}\cdot {\dot{X}}_{p-0},$$

denotes the velocity of the platform connection point corresponding to the j-th leg, as expressed in the mobile platform reference frame. This velocity is influenced by the generalized motion of the platform and is critical for computing the actuator rate required to maintain the desired trajectory.

By combining the actuator velocity mapping with the geometric relation between platform points and generalized motion, the complete Jacobian expression becomes

(11) $$\dot{q}=J_{IA}\cdot J_{IIA}\cdot {\dot{X}}_{p-0},$$

This form relates the linear velocity of each actuator with the velocity vector of the mobile platform via the analytical Jacobians.

The STR architecture involves three active prismatic joints; the first Jacobian matrix is

(12) $$J_{IA}=\left[\begin{array}{ccc} {\vec{u}}_{1}^{T} & 0 & 0\\ 0 & {\vec{u}}_{2}^{T} & 0\\ 0 & 0 & {\vec{u}}_{3}^{T} \end{array}\right],$$

where each row corresponds to one of the three actuator legs, and ${\vec{u}}_{i}\in R^{3}$ is the unit direction vector of the prismatic actuator axis and can be obtained by

$${\vec{u}}_{i}=\frac{L_{i}}{q_{i}},$$

(13) $$\dot{q_{i}}=\dot{z}+\omega \times r_{i}$$

where $\omega =\left[\begin{array}{c} \omega_{x}\\ \omega_{y}\\ 0 \end{array}\right]$; $r_{i}=R\cdot P_{i}$.

For parallel mechanisms with 3 kinematic chains, the second Jacobian matrix in Equation (11) is calculated as the following:

(14) $$J_{IIA}={\left[\begin{array}{cc} I_{3\times 3} & S\left(\omega \right)\cdot R\cdot P_{1}\\ I_{3\times 3} & S\left(\omega \right)\cdot R\cdot P_{2}\\ I_{3\times 3} & S\left(\omega \right)\cdot R\cdot P_{3} \end{array}\right]}_{9\times 6}$$

where $I_{3\times 3}$ denotes the identity matrix. *S*(*ω*) designates the 3 × 3 symmetric matrix defined by the vector $\omega =\left[\begin{array}{c} \omega_{x}\\ \omega_{y}\\ 0 \end{array}\right]$, $S\left(\omega \right)=\left[\begin{array}{ccc} 0 & 0 & \omega_{y}\\ 0 & 0 & -\omega_{x}\\ -\omega_{y} & \omega_{x} & 0 \end{array}\right]$. $R=R_{x}\left(\varphi \right)R_{y}\left(\theta \right)$ is the full rotation matrix for the platform and *P_i_* are the position vectors of attachment points on the platform.

### 2.4. Inverse Dynamics

Inverse dynamics for the proposed parallel mechanisms with three degrees of freedom aims to determine the forces and torques applied by the prismatic actuators necessary to follow a desired trajectory. The dynamic model is derived using the principle of virtual work, applied to both the moving platform and each kinematic chain (leg) [28,29]. The forces and torques acting at the center of mass of the mobile platform are described by the same vector structure:

(15) $$F_{p}=\left[\begin{array}{c} {\hat{f}}_{p}\\ {\hat{n}}_{p} \end{array}\right]=\left[\begin{array}{c} f_{e}+m_{p}\cdot g-m_{p}{\cdot \dot{V}}_{p}\\ n_{e}+I_{pB}\cdot {\dot{\omega }}_{p}-\omega_{p}\times \left(I_{pB}\cdot \omega_{p}\right) \end{array}\right]$$

where F_p_ is the vector of forces and resulting moments on the moving platform;

F_e_ is the external force exerted at the center of mass;
n_e_ is the external moment applied at the articulation between the platform and leg;
m_p_ is platform mass;
${\dot{V}}_{p}$ is linear acceleration of the center of gravity of the platform;
I_pB_ is the platform’s inertia matrix, expressed in the base coordinate system [kg·m^2^];
ω_p_ is the angular velocity of the moving platform.

The inertia matrix of the mobile platform is transformed from the local platform frame to the base frame using

(16) $$I_{pB}=R_{pB}\cdot I_{pP}\cdot R_{BP},$$

where *R_pB_* and *R_BP_* represent orthogonal transformation matrices;

*I_pP_* is the inertia matrix of the platform in a local frame.

All legs are identical, and for the force and moment computation, we will adapt a single equation for all three legs, *i* ∈ {1, 2, 3}.

Each kinematic chain consists of two rigid bodies: an actuator cylinder and a piston-type connecting rod. The inertial effects of these components must be separately evaluated to correctly model the dynamic behavior of the mechanism, so the inertial force and moment expressions for each of the two elements of the leg, “i”, must be written in their local reference frames {Pi}.

Assuming that the only external force is gravity, the resultant forces and moments acting on the actuator cylinder are given by:

(17) $$F_{1i}=\left[\begin{array}{c} {\hat{f}}_{1i}\\ {\hat{n}}_{1i} \end{array}\right]=\left[\begin{array}{c} m_{1}\cdot g\cdot R_{B1i}-m_{1}{\cdot \dot{V}}_{1i}\\ I_{1i}\cdot {\dot{\omega }}_{1i}-\omega_{1i}\times \left(I_{1i}\cdot \omega_{1i}\right) \end{array}\right],$$

where m_1_ is mass of the actuator cylinder of the same legs;

V_i1_ is linear acceleration of the actuator cylinder;
ω_1i_ is angular velocity and acceleration;
I_1i_ is the moment of inertia of the cylinder concerning the local reference frame {P_i_};
R_B1i_ is rotation matrix from global {B_i_} to local frame.

The second rigid body of the kinematic chain, the piston rod, is affected by inertial forces and gravitational loading. Its dynamic model is expressed as

(18) $$F_{2i}=\left[\begin{array}{c} {\hat{f}}_{2i}\\ {\hat{n}}_{2i} \end{array}\right]=\left[\begin{array}{c} m_{2}\cdot g\cdot R_{B2i}-m_{2}{\cdot \dot{V}}_{2i}\\ I_{2i}\cdot {\dot{\omega }}_{2i}-\omega_{2i}\times \left(I_{2i}\cdot \omega_{2i}\right) \end{array}\right],$$

where m_2_ is mass of the actuator piston of the same legs;

V_i2_ is linear acceleration of the actuator piston;
ω_2i_ is angular velocity and acceleration;
I_2i_ is the moment of inertia of the piston concerning the local reference frame {P_i_};
R_B2i_ is rotation matrix from global {B_i_} to local frame.

According to the virtual work principle, the total virtual work produced by all forces and moments in the system must be zero:

(19) $$\delta x_{p}^{T}\cdot F_{p}+\delta q_{i}^{T}\cdot \tau +\sum_{i=1}^{3}\left(\delta x_{1i}^{T}\cdot F_{1i}+\delta x_{2i}^{T}\cdot F_{2i}\right)=0,$$

where x_p_ is the position vector of the moving platform;

F_p_ is the vector of external forces and moments on the moving platform;
q_i_ is the vector of lengths of each leg;
τ is the vector of the torques applied to the revolute joints;
F_1i_ and F_2i_ are the resultant forces and moments on actuators’ components in each leg;
δx_1i_ and δx_2i_ represent the positions of each actuator component (cylinders and pistons), expressed in their respective frames of reference.

This method enables the derivation of actuator torques based on the external forces and moments acting on each body within the mechanism which consists of three identical kinematic chains.

To express the virtual displacements δx as functions of generalized coordinates, the following relations are used:

(20) $$\delta q=J_{p}\cdot \delta x_{p}=J_{p}\cdot J_{x_{p}}\cdot \omega_{p},$$

(21) $$\delta x_{1i}=J_{1i}\cdot \delta x_{p}=J_{1i}\cdot J_{x_{p}}\cdot \omega_{p}$$

(22) $$\delta x_{2i}=J_{2i}\cdot \delta x_{p}=J_{2i}\cdot J_{x_{p}}\cdot \omega_{p}$$

Substituting Equations (20)–(22) into (19) yields the generalized motion equation for the parallel robot. It is important to note that the torques acting on the system are expressed relative to the center of mass of each rigid body within the kinematic chains. However, if an external torque is applied at a point other than the center of mass, for instance, at the actuator mounting or joint locations, it must first be transformed into an equivalent torque referenced about the center of mass before being introduced into the dynamic motion equations:

(23) $$\omega_{p}^{T}\cdot \left[J_{p}^{T}\cdot J_{x_{p}}^{T}\cdot \tau +J_{x_{p}}^{T}\cdot F_{p}+\sum_{i=1}^{3}\left(J_{1i}^{T}\cdot F_{1i}\cdot J_{x_{p}}^{T}+J_{2i}^{T}\cdot F_{2i}\cdot J_{x_{p}}^{T}\right)\right]=\;0,$$

Substituting Equations (15) and (16) into (23) and solving for τ leads to the expression of required joint torques for actuation:

(24) $$\tau =-{\left(J_{p}^{T}\cdot J_{x_{p}}^{T}\right)}^{-1}\cdot \left[J_{x_{p}}^{T}\cdot F_{p}+\sum_{i=1}^{3}\left(J_{1i}^{T}\cdot F_{1i}\cdot J_{x_{p}}^{T}+J_{2i}^{T}\cdot F_{2i}\cdot J_{x_{p}}^{T}\right)\right]$$

### 2.5. Multi-Criteria Evaluation via AHP

To objectively compare the performance of the four configurations considered in this study, a multi-criteria decision-making process was implemented using the Analytic Hierarchy Process (AHP) [30]. This method is particularly suitable for engineering applications involving multiple evaluation criteria and allows combining both quantitative simulation outputs and qualitative expert judgment.

Simulation data provide measurable indicators for performance, such as tracking error, actuator effort, mechanical range, or control responsiveness, which may not be equally weighted in all contexts. AHP allows the integration of these diverse criteria into a single composite score, enabling rational selection based on weighted priorities.

Four performance criteria were defined based on their relevance to robotic rehabilitation applications, precision, speed, effort, and operational workspace:

C1—Control Accuracy: The ability of the robot to follow reference trajectories, expressed as the value of the tracking error between commanded and measured actuator positions. Lower values indicate more efficient designs.

C2—Velocity Response: The minimum velocity achieved by actuators during simulation, indicative of low energy needed. Lower values indicate more efficient designs.

C3—Force Efficiency: The average force required by each actuator to execute prescribed movements while subjected to external loads (simulating patient interaction). Lower values indicate more efficient designs.

C4—Mechanical Range: The spatial traversed by the mobile platform during simulation, reflecting the system’s ability to support a wide range of rehabilitation exercises.

The relative importance of each criterion was determined through pairwise comparisons, based on expert judgment and references in the literature. The priority weights were derived using the Saaty scale and processed into a comparison matrix [31].

The resulting weights were as follows: w_1_ = 0.30; w_2_ = 0.20; w_3_ = 0.30; w_4_ = 0.20. These weights reflect the assumption that precision and force economy are equally critical in rehabilitation scenarios, while speed and range are important but slightly less dominant.

To validate the logical consistency of the pairwise matrix, the Consistency Ratio (CR) was calculated as follows:

(25) $$CR\;=\;((\lambda_{max}\;-\;n)\;/\;(n\;-\;1))\;/\;RI,$$

where λ_max_ is the maximum eigenvalue of the pairwise comparison matrix; n is the number of criteria; and RI is the Random Index, a known constant that depends on n (for n = 4, RI = 0.90) [23].

If CR < 0.10, the consistency of the judgments is considered acceptable [32,33]. In this case, the computed CR was below the threshold.

To bring all simulation results to a common scale, the normalization method was used. For cost-type criteria (C1, C2, and C3), the normalized score was calculated using the formula:

(26) $$r_{i}=\frac{\left(X_{max}-X_{min}\right)}{\left(X_{max}-X_{i}\right)}\;,$$

while for benefit-type criteria (C4), the formula was as follows:

(27) $$r_{i}=\frac{\left(X_{i}-X_{min}\right)}{\left(X_{max}-X_{min}\right)}\;,$$

where *X_i_* is the value for a given configuration for those criteria; *X_min_* and *X_max_* are the lowest and highest values for that criterion among all configurations.

This ensured that each score lies between 0 and 1, allowing meaningful comparisons across criteria with different units or scales.

Each configuration’s overall AHP score was calculated using the weighted sum method:

(28) $${AHP}_{Score}=w_{1}\cdot r_{1}+w_{2}\cdot r_{2}+w_{3}\cdot r_{3}+w_{4}\cdot r_{4}$$

where w_1_ to w_4_ are the weights of the four criteria; r_1_ to r_4_ are the normalized scores for each criterion.

The configuration with the highest total score is considered the most suitable design under the selected evaluation criteria.

### 2.6. Simulations Setup Models

The proposed simulations aim to verify the following aspects:

- The system’s ability to reach all required points in the workspace.
- The accuracy of movements and the system’s ability to automatically adjust according to the patient’s needs.
- The forces required by the actuators to follow predefined trajectories, while the mobile platform is subjected to external forces applied by the patient.
- The velocities required by the actuators to follow reference trajectories.

**Figure A1:**
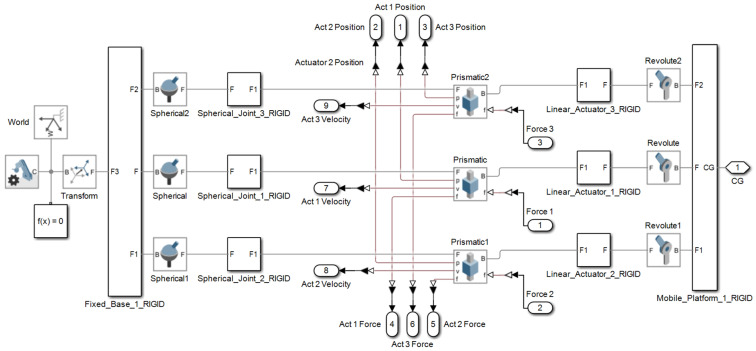
Simulation model—3STR mechanism.

**Figure A2:**
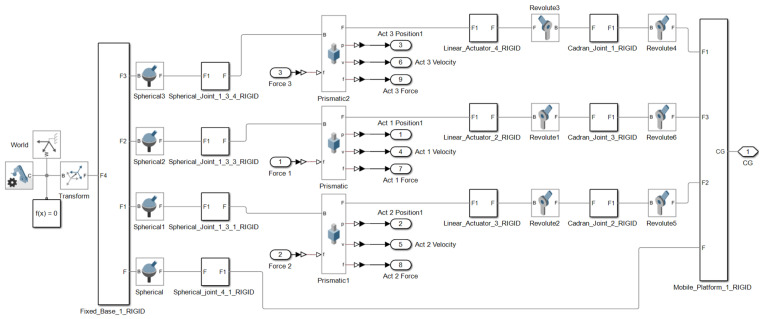
Simulation model—3STC mechanism.

**Figure A3:**
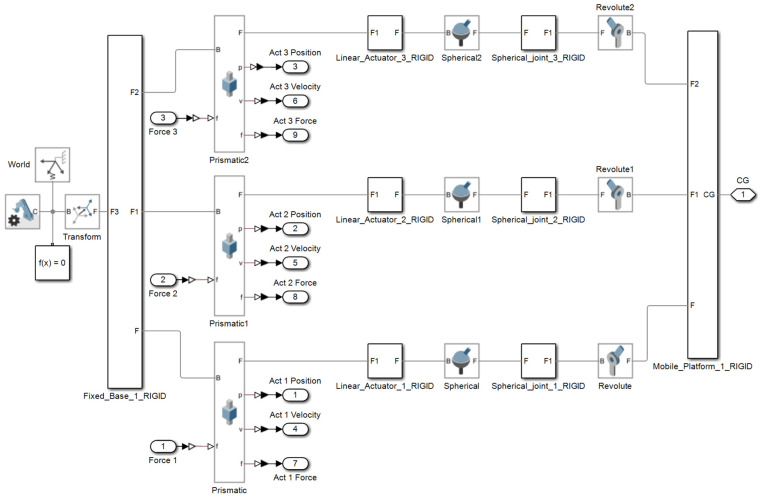
Simulation model—3TSR mechanism.

**Figure A4:**
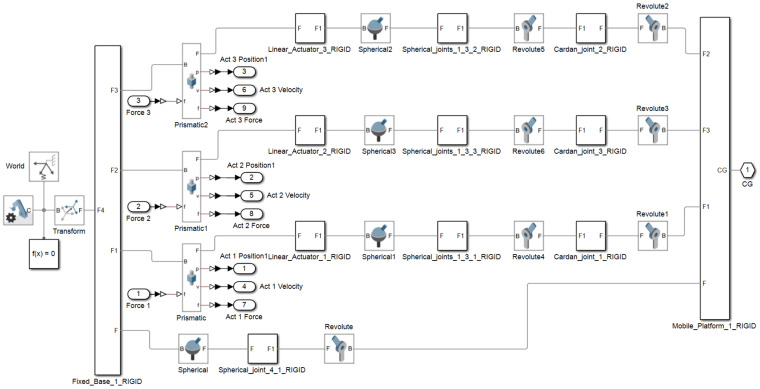
Simulation model—3TSC mechanism.

**Figure A5:**
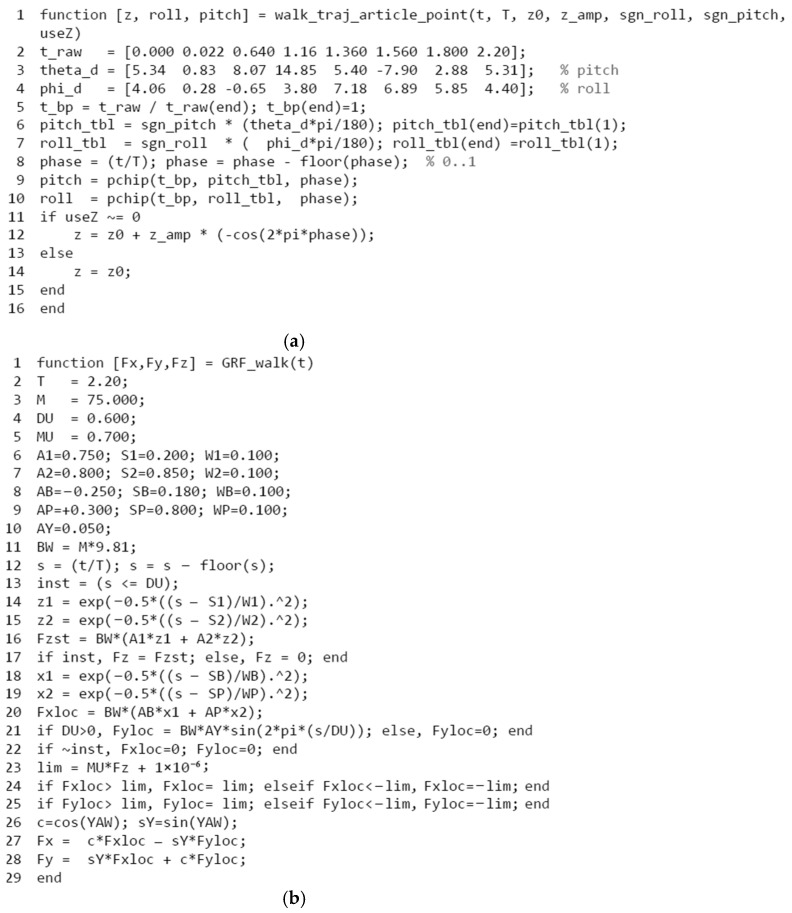
Gait coded in MATLAB. (**a**) Gait motion generator module; (**b**) Forces applied by the patient during gait, simulator module.

**Figure A7:**
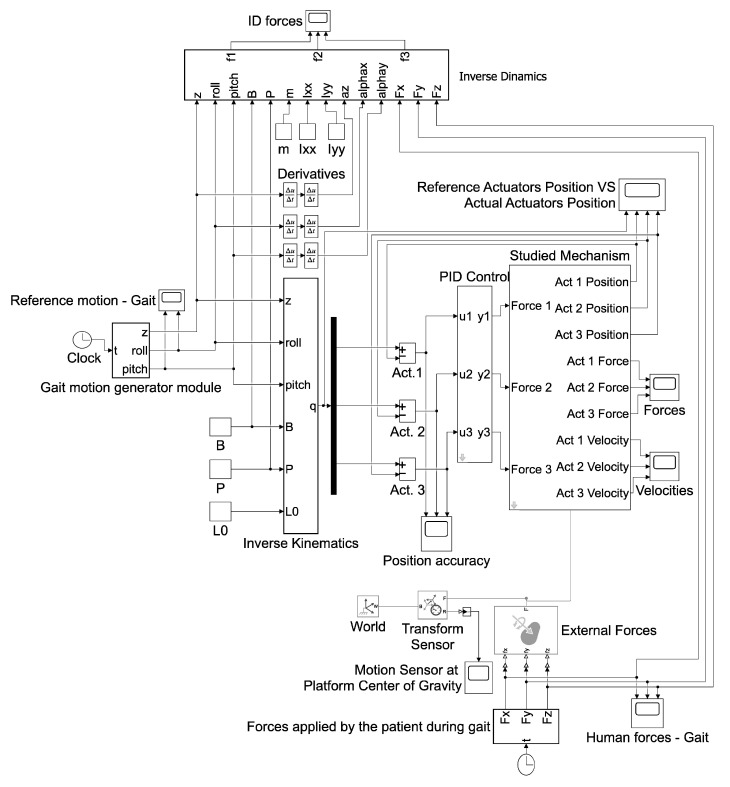
Complete Simulink model.

To perform simulations in various scenarios and especially to enable a comparative analysis of the selected configurations, the following steps were carried out:

- Design of the CAD geometric models with the relevant parameters, as shown in Figure 1, Figure 2, Figure 3 and Figure 4.
- Build of the simulation models for each of the studied mechanisms from the exported CAD and.xml files in the MATLAB-Simulink environment, as illustrated in Appendix A Figure A1, Figure A2, Figure A3 and Figure A4.
- Realization of a gait motion generator module for the mobile platform, in accordance with article [34], which outputs the desired platform pose as a time-periodic walking cycle (see Appendix A Figure A5a).
- Programming of a module that simulates the forces applied by the patient during gait-rehabilitation exercises, at the center of gravity of the mobile platform. The script generates the components of the external reaction forces, from the gait, on the mobile platform (see Appendix A Figure A5b).
- Implementation of the inverse kinematics and inverse dynamics algorithms as MATLAB functions, like in Appendix A—Figure A6.
- Development of a closed-loop control algorithm based on three PD (Proportional–Derivative) controllers, one per actuator, so that each actuator is commanded with the force required for the mobile platform to track the gait motion prescribed, even in the presence of patient-induced forces (Appendix A Figure A7). The force sensors monitor the movements in real time and adjust the actuators to maintain the predefined trajectory, under safe conditions for the patient.

#### Simulation Inputs

**Figure 5:**
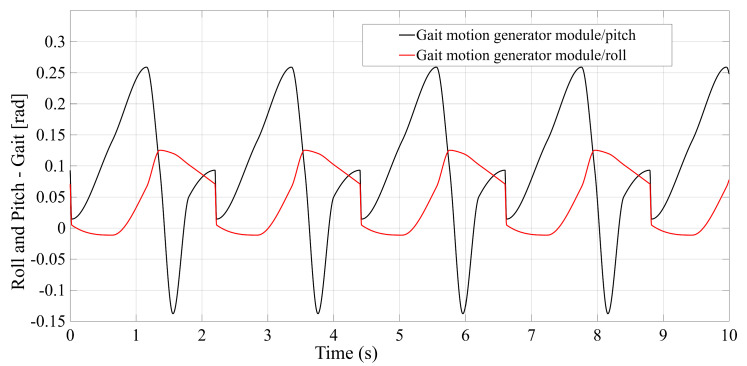
Reference motion—Gait: roll and pitch.

**Figure 6:**
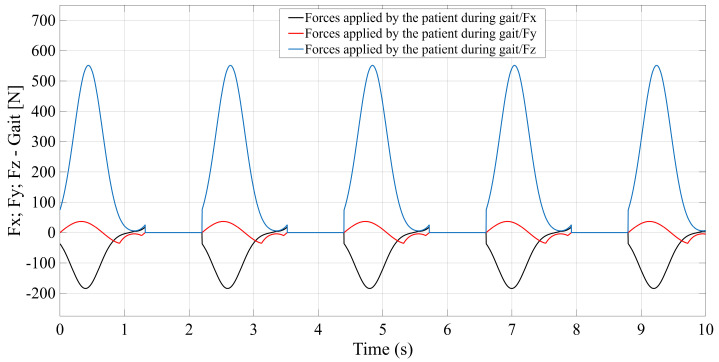
Human forces—Gait: Fx, Fy, Fz.

**Figure A8:**
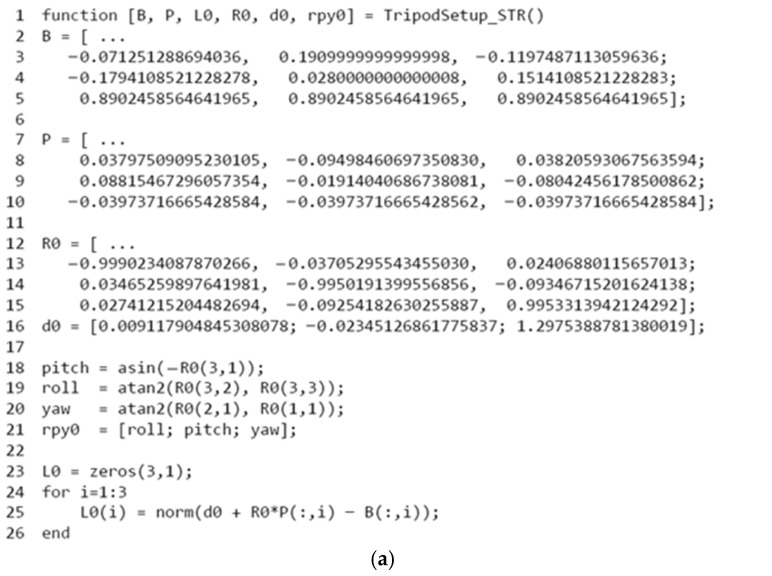

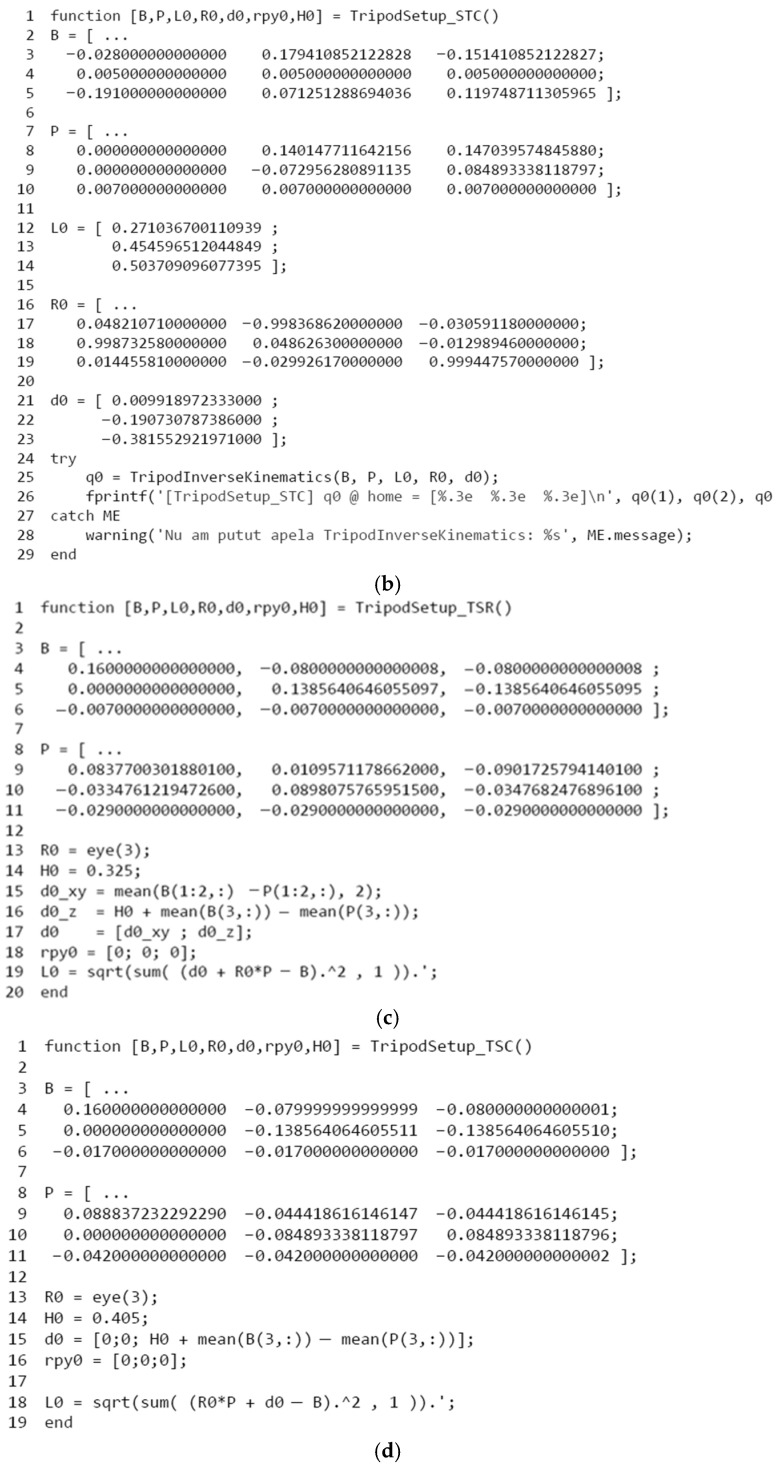
Geometric properties of studied mechanisms. (**a**) STR; (**b**) STC; (**c**) TSR; (**d**) TSC.

To evaluate the performance of each configuration (STR, STC, TSR, TSC), simulations were conducted in MATLAB-Simulink under identical input conditions. The common input data are the platform motion trajectories (see Figure 5) and the forces applied by the patient (see Figure 6). For each type of mechanism studied, all geometric parameters and physical properties are extracted from CAD into a MATLAB data file, from which those involved in the application of the inverse kinematics and dynamics methods are presented in Appendix A Figure A8. Mechanical limitations: Spherical joints: ±16 [°]; ±20 [°]; rotational joints: ±7 [°]; Cardan joints: ±7 [°]; Actuator full stroke: 0.15 [m].

## 3. Results

### 3.1. Numerical Simulation Results

**Figure 7:**
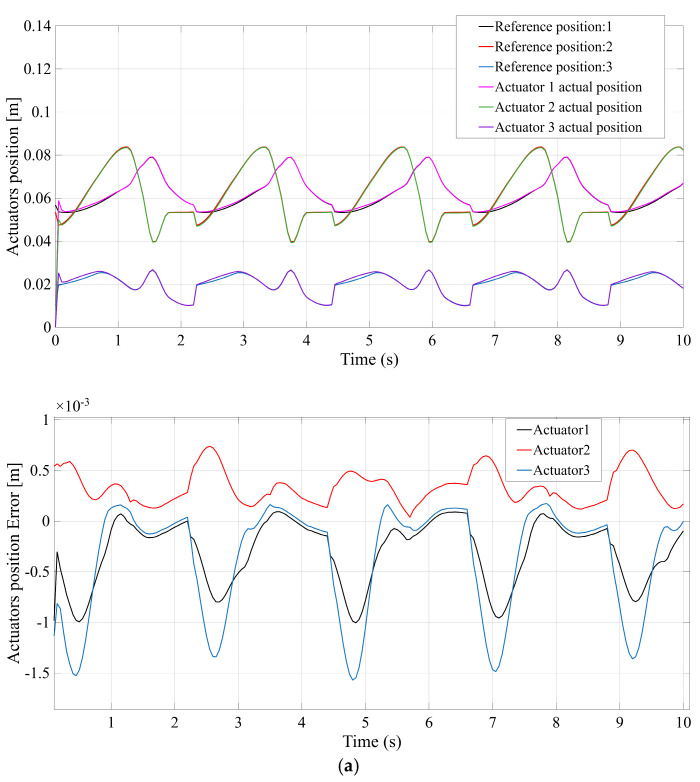

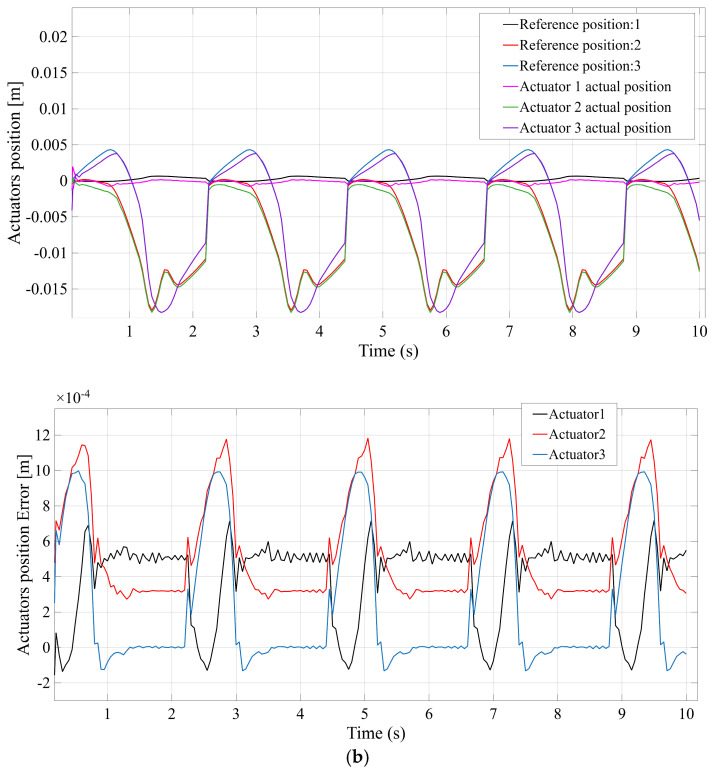

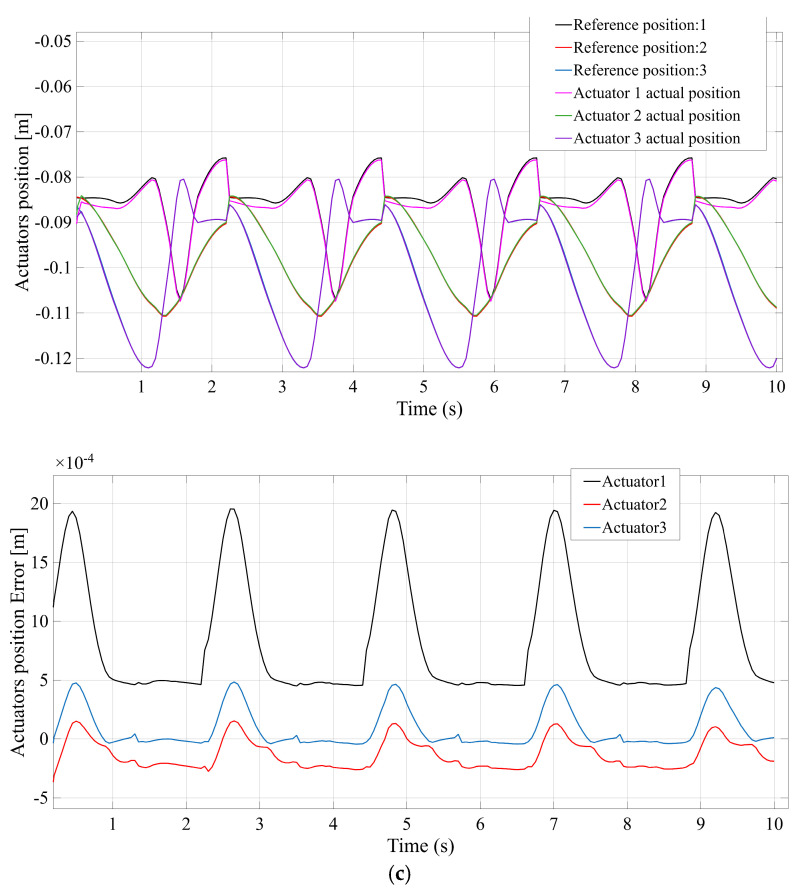

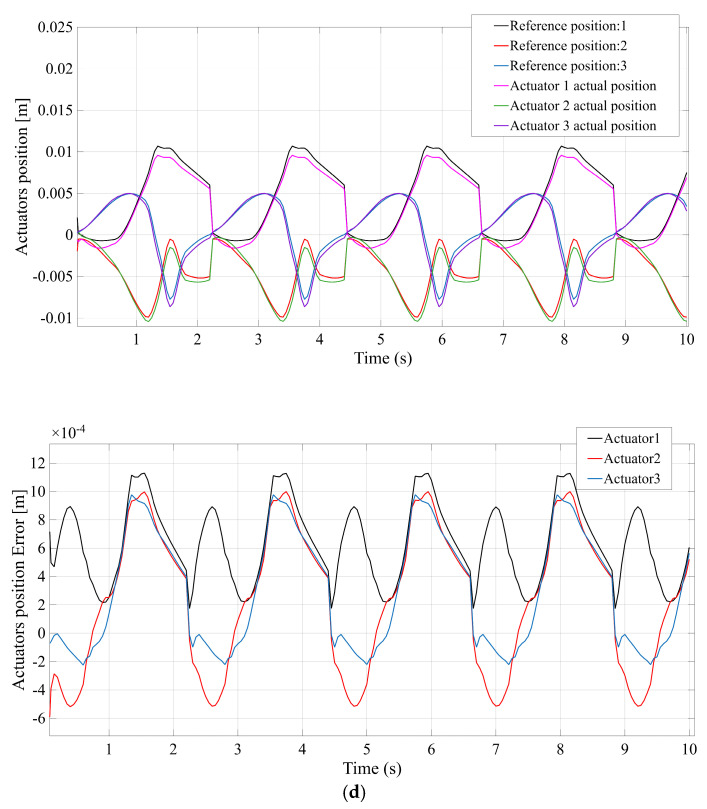
Actuator reference vs. actual position and resulting position error. (**a**) STR; (**b**) STC; (**c**) TSR; (**d**) TSC.

The control accuracy of each robotic configuration was assessed by evaluating the tracking error of the actuator’s position. This error is defined as the difference between the commanded or reference position for actuators and the actual or measured position of the actuators. Figure 7 compares the actuator position tracking error for each mechanism, with Figure 7a–d corresponding to the STR, STC, TSR, and TSC configurations, respectively. All four systems are able to follow the prescribed gait trajectory closely under PD control, but clear differences in precision emerge. Figure 7a (STR) shows that the STR design maintains only moderate accuracy—the prismatic actuators exhibit small steady-state errors and minor oscillations, reaching a peak error on the order of a few millimeters. Figure 7b (STC), in contrast, demonstrates improved tracking accuracy: the STC mechanism’s actuators achieve near-perfect tracking, with a maximum error of roughly one millimeter (substantially lower than STR). This higher precision is attributable to the stabilizing effect of the additional central spherical joint, which helps constrain unwanted platform motion. Figure 7c (TSR) shows results for the TSR configuration, which are intermediate—the tracking error remains low (on the order of 1–2 mm) but slightly higher than the STC case. Finally, Figure 7d (TSC) indicates that the TSC design also attains high control accuracy, comparable to STC, with only minimal error throughout the motion. In summary, while all mechanisms achieve the commanded positions, the STC and TSC configurations yield the smallest actuator position errors, reflecting superior control accuracy, whereas STR (and to a lesser extent TSR) exhibits the largest deviation between commanded and actual positions.

**Figure 8:**
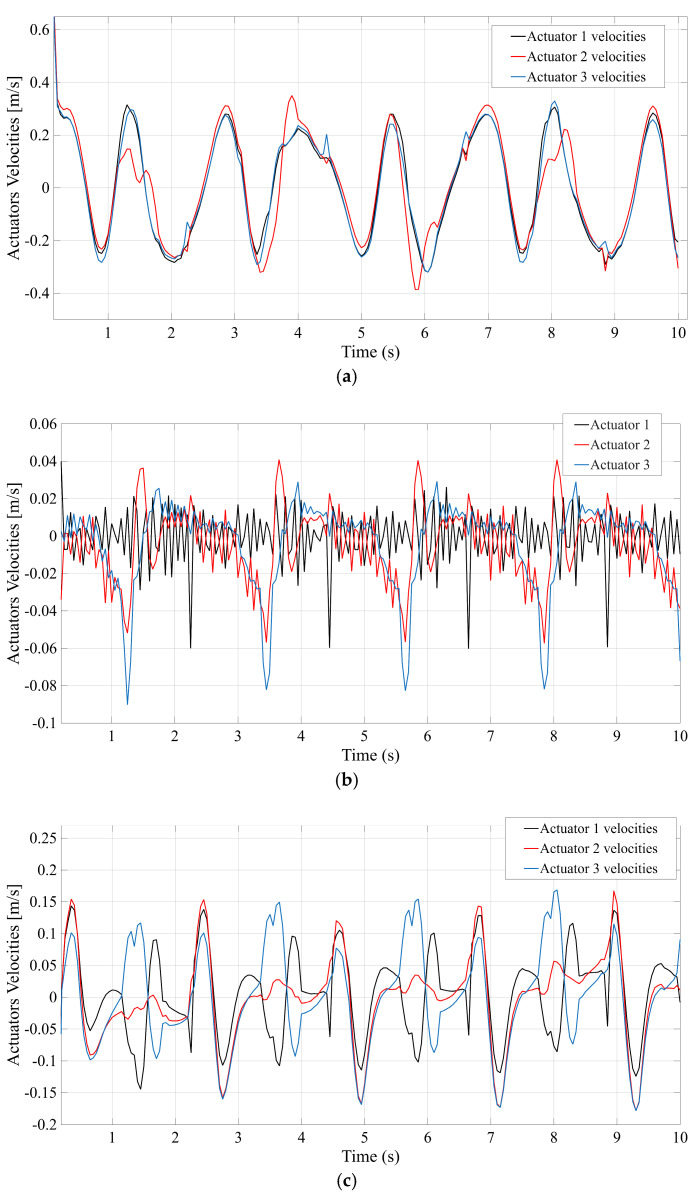

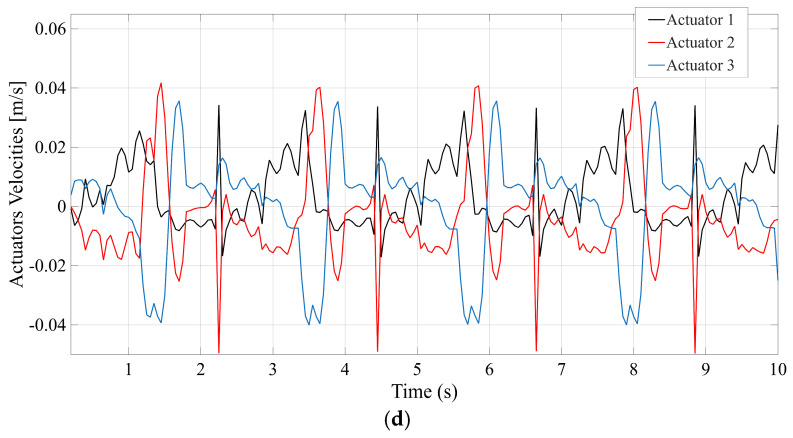
Actuators’ velocities. (**a**) STR; (**b**) STC; (**c**) TSR; (**d**) TSC.

Figure 8 illustrates the actuator velocity response for each configuration during the gait cycle, again with Figure 8a–d corresponding to STR, STC, TSR, and TSC. The plots highlight the speed at which each prismatic actuator must extend and retract to track the reference motion. In Figure 8a (STR), the STR mechanism’s actuators reach the highest peak velocity among the four designs. The plot shows a rapid oscillatory velocity profile with a maximum value of approximately 0.6 m/s (indicating that STR demands the fastest actuator movements to replicate the gait trajectory). Figure 8b (STC) reveals a noticeably lower velocity demand for the STC system—the actuators operate more gently, with peak speeds around 0.4 m/s. This reduction in required speed suggests that the central passive spherical joint in STC allows motion to be distributed more efficiently, so each actuator undergoes a smaller length change for the same platform movement. The TSR configuration (Figure 8c) shows a velocity profile that is somewhat lower than STR but still higher than the designs with a central spherical coupling. TSR’s actuators reach roughly 0.5 m/s at peak, reflecting that without a central support, they must compensate with faster action than in STC. Meanwhile, Figure 8d (TSC) indicates that the TSC mechanism achieves a performance similar to STC—its required actuator velocities are relatively low (peaking at roughly 0.45 m/s). Overall, the mechanisms containing the additional central spherical joint (STC and TSC) manage to fulfill the gait motion with more modest actuator speeds, whereas STR and TSR demand higher peak velocities to accomplish the same task.

**Figure 9:**
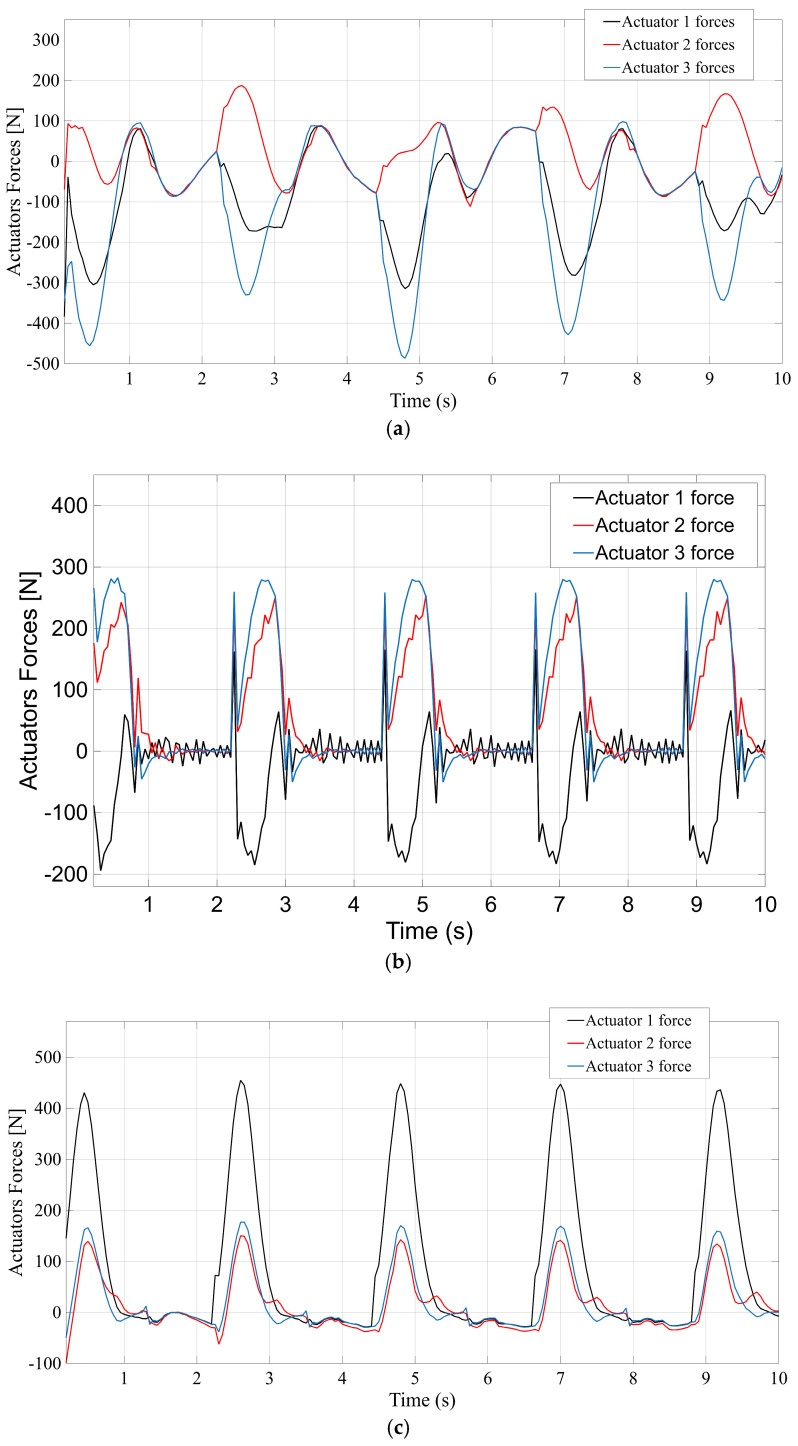

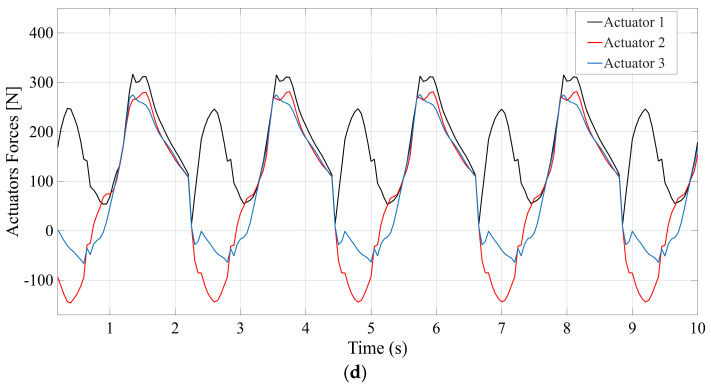
Actuators’ forces. (**a**) STR; (**b**) STC; (**c**) TSR; (**d**) TSC.

Figure 9 compares the forces developed by the actuators in each mechanism when executing the gait trajectory under load. Figure 9a–d correspond to the STR, STC, TSR, and TSC designs, respectively, and each plot reflects the actuator force as a function of time (including the effect of simulated patient interaction forces applied to the platform). In the STR system (Figure 9a), the actuators experience the largest forces among the four configurations. The plot shows that STR’s actuators must generate a peak force on the order of 100 N to maintain the prescribed motion against the external disturbances. This relatively high force demand can be attributed to the STR architecture lacking any auxiliary support—the three legs alone bear the full load of the platform and applied forces, leading to higher stresses per actuator. Figure 9b (STC) demonstrates a significant reduction in peak actuator force for the STC design. The maximum force for STC is only about 60 N, markedly lower than STR. The presence of the central passive spherical coupling in STC allows part of the load to be distributed through the platform’s center, relieving the actuators from carrying the entire weight and external force by themselves. The TSR mechanism (Figure 9c) shows intermediate behavior: its actuator forces reach around 90 N at maximum, lower than STR but still considerably above the STC case. TSR’s spherical joint in the leg improves force distribution slightly compared to STR, but without a central support it cannot reduce the load on actuators as effectively as STC does. Finally, the TSC configuration (Figure 9d) achieves a low actuator force profile comparable to STC. The peak force in TSC (roughly 70 N) is much lower than in the absence of a central joint, underscoring that the combined translational–spherical–cardan design with a central support efficiently handles the external loading. In summary, the two architectures incorporating a central spherical support (STC and TSC) exhibit superior force efficiency, requiring significantly smaller actuator forces to perform the motion, whereas the STR and TSR designs see substantially higher force peaks due to their actuators solely resisting gravity and patient-induced forces.

**Figure 10:**
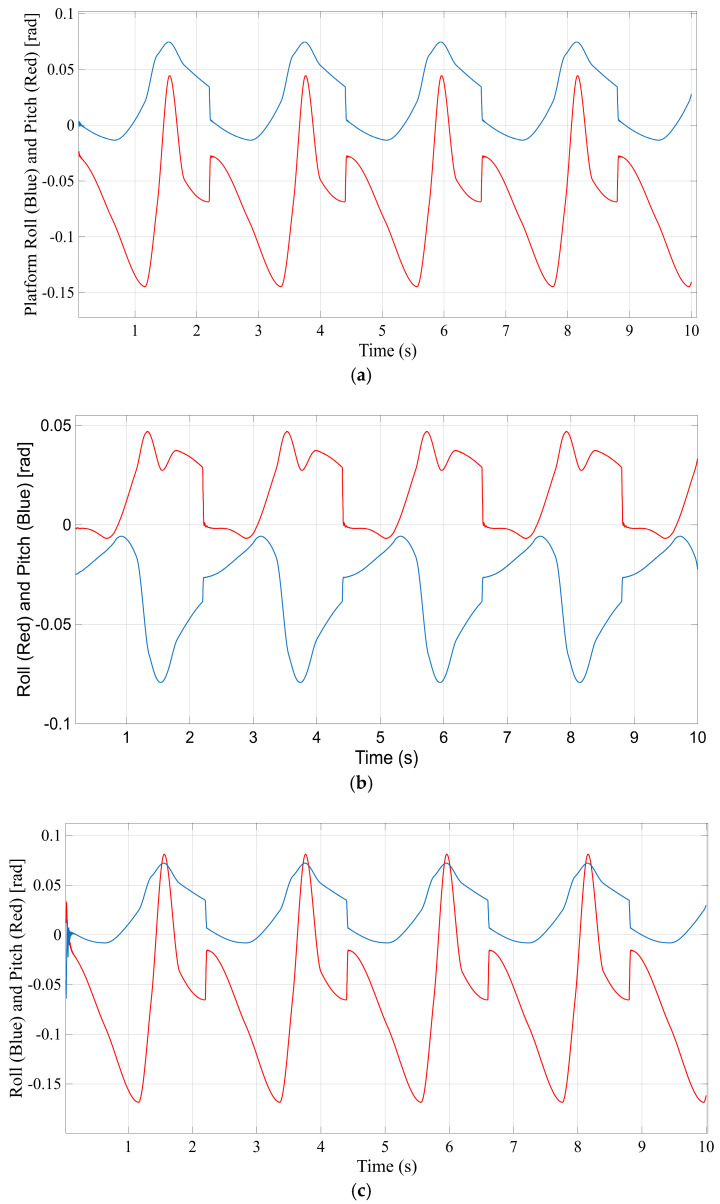

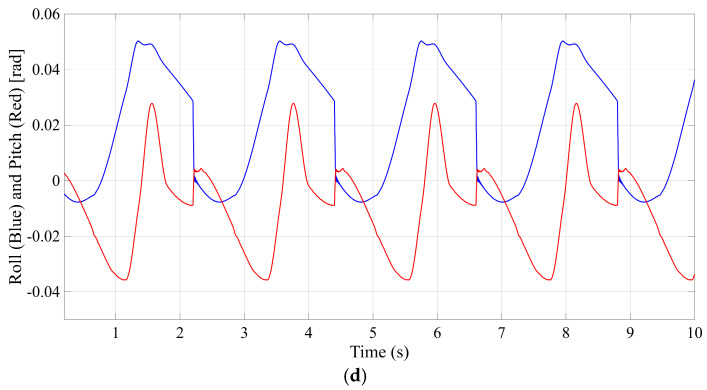
Position of center of gravity (CG) of the mobile platform. (**a**) STR; (**b**) STC; (**c**) TSR; (**d**) TSC.

Figure 10 presents the motion of the moving platform’s center of gravity (CG) for each configuration throughout the gait cycle. Figure 10a–d correspond to the STR, STC, TSR, and TSC mechanisms, respectively, and plot the vertical displacement of the platform’s CG over time. This comparison reflects the mechanical range of motion that each design permits under the given actuation. Figure 10a (STR) shows that the STR mechanism’s platform achieves a noticeable vertical movement during the exercise—the CG oscillates upward and downward with a peak-to-peak amplitude of several centimeters (approximately 50 mm of total displacement from the lowest to highest point). This substantial platform travel is expected, since STR’s three actuators actively lift and lower the platform without additional constraints (aside from the leg geometry). The STC configuration (Figure 10b), on the other hand, displays a markedly smaller net displacement of the platform’s center. The CG in STC remains almost fixed in space, with only a very slight vertical motion (on the order of a few millimeters). This is because the central spherical joint connecting the platform to the base in STC constrains pure translation—the platform primarily rotates about the central pivot, resulting in minimal vertical excursion of its center of gravity. Similarly, the TSR mechanism (Figure 10c) produces a moderate platform motion; its CG trace shows an amplitude comparable to STR (around 50 mm). Like STR, the TSR design can translate the platform vertically using its three actuated legs (since no central link restricts the motion), so the full commanded displacement is realized. In contrast, the TSC design (Figure 10d) behaves like STC with respect to CG movement—the platform’s center remains nearly stationary, exhibiting only a very small movement. The TSC architecture’s central supporting joint effectively anchors the platform’s center, allowing mainly rotational movement. In summary, STR and TSR permit a larger mechanical range of motion (greater vertical platform displacement), whereas STC and TSC constrain the platform’s translation, resulting in a much smaller CG travel. All four mechanisms delivered the intended rotational movements (roll and pitch as required by the gait pattern), but the total spatial displacement of the platform differs due to their kinematic constraints. This distinction is important because a larger platform motion range (as seen in STR/TSR) could be advantageous for certain exercises, while a more constrained motion (STC/TSC) might prioritize stability over range.

To objectively compare the designs across these multiple criteria, we next perform a multi-criteria decision analysis (AHP).

### 3.2. Multi-Criteria Evaluation Using the Analytic Hierarchy Process Based on Simulation Results

To quantitatively rank the four candidate mechanisms, an Analytic Hierarchy Process (AHP) analysis was performed using the above simulation results. Four performance criteria were considered, corresponding to the key outcomes in Figure 7, Figure 8, Figure 9 and Figure 10: (C1) Control Accuracy, measured by the maximum actuator tracking error; (C2) Velocity Response, measured by the maximum actuator velocity; (C3) Force Efficiency, measured by the maximum actuator force; and (C4) Mechanical Range, measured by the maximum displacement of the platform’s center of gravity. The maximum observed values for each criterion, extracted from Figure 7, Figure 8, Figure 9 and Figure 10, are summarized below for the four configurations:

STR: C1 = 3 mm (max error); C2 = 0.6 m/s (max velocity); C3 = 100 N (max force); C4 = 50 mm (max CG displacement).
STC: C1 = 1 mm; C2 = 0.4 m/s; C3 = 60 N; C4 = 5 mm.
TSR: C1 = 2.5 mm; C2 = 0.5 m/s; C3 = 90 N; C4 = 50 mm.
TSC: C1 = 1.5 mm; C2 = 0.45 m/s; C3 = 70 N; C4 = 5 mm.

In the context of decision analysis, lower values are preferable for criteria C1, C2, and C3 (since smaller error, lower speed, and less force indicate better performance), whereas a higher value is preferable for C4 (since a larger motion range is beneficial). Thus, to combine these metrics, the raw values are first normalized to dimensionless scores on a common scale (0 to 1) using Equations (26) and (27). For the three cost-type criteria (C1, C2, C3), normalization is performed by subtracting each value from the worst (highest) value in that criterion range, so that the best (lowest) performer receives a normalized score of 1.0 and the worst receives 0. For the benefit-type criterion (C4), normalization is performed by dividing by the highest value, so that the design with the largest platform displacement receives a score of 1.0. The normalized scores for all four criteria after this transformation, are as follows:

STR: r1 = 0.00; r2 = 0.00; r3 = 0.00; r4 = 1.00.
STC: r1 = 1.00; r2 = 1.00; r3 = 1.00; r4 = 0.00.
TSR: r1 = 0.25; r2 = 0.50; r3 = 0.25; r4 = 1.00.
TSC: r1 = 0.75; r2 = 0.75; r3 = 0.75; r4 = 0.00.

Finally, the overall AHP priority score for each configuration is obtained by taking a weighted sum of these normalized criterion scores. Based on expert judgment and the literature (see Section 2), the weights assigned were w1 = 0.30 for Control Accuracy, w2 = 0.20 for Velocity Response, w3 = 0.30 for Force Efficiency, and w4 = 0.20 for Mechanical Range. These weights reflect an emphasis on precise motion and low actuator effort (each contributing 30% of the decision importance), while still valuing speed and range of motion to a lesser extent (20% each). Using Equation (28) (the weighted sum formula), the composite AHP score for each design was calculated as follows:

STR: Score = 0.30·(0.00) + 0.20·(0.00) + 0.30·(0.00) + 0.20·(1.00) = 0.20.
STC: Score = 0.30·(1.00) + 0.20·(1.00) + 0.30·(1.00) + 0.20·(0.00) = 0.80.
TSR: Score = 0.30·(0.25) + 0.20·(0.50) + 0.30·(0.25) + 0.20·(1.00) = 0.45.
TSC: Score = 0.30·(0.75) + 0.20·(0.75) + 0.30·(0.75) + 0.20·(0.00) = 0.60.

These results indicate that STC achieves the highest overall score (0.80) among the four evaluated configurations. In other words, when considering all criteria together, the spherical–translational–cardan design with an added central spherical coupling is predicted to be the most effective solution for rehabilitation therapy needs. The second-ranked design is TSC (score 0.60), which also includes the central stabilizing joint and exhibits a strong all-around performance. The TSR configuration scores 0.45, coming in third—its performance is moderate but it lacks the special advantages conferred by the central coupling. Finally, STR ranks fourth with a composite score of 0.20, primarily because its poorer tracking precision and higher force/velocity demands outweighed its advantage in platform motion range. It is worth noting that the two mechanisms with a central spherical support (STC and TSC) decisively outperform the others in this quantitative ranking, reflecting the importance of stability and reduced actuator loads in the weighted criteria. The AHP analysis thus provides a rational basis for design selection: STC emerges as the top candidate for a parallel rehabilitation robot, given the specified weighting of performance priorities.

## 4. Discussion

The comparative simulation results and AHP evaluation highlight significant trade-offs between the four parallel robot configurations and offer insight into the optimal design for rehabilitation therapy. In general, the mechanisms augmented with a central spherical joint (STC and TSC) demonstrated superior performance in several key aspects. They achieved the highest control accuracy, with tracking errors roughly half those of the other designs, and required substantially lower actuator forces and velocities to realize the same patient motion. These findings are consistent with theoretical expectations and prior research: adding a passive supporting linkage increases the platform’s stability and effectively distributes loads, thereby reducing the burden on any single actuator. The improved precision observed in the STC and TSC variants can be attributed to their increased structural rigidity and constraint of extraneous motion. In effect, the central spherical coupling acts as a stabilizing pivot, preventing unintended platform translations and vibrations, which in turn leads to tighter trajectory tracking. This observation aligns with earlier studies that noted that spherical joint architectures can enhance kinematic control and mimic natural joint motion more closely in rehabilitation robots.

Another important outcome of this study is the influence of kinematic architecture on actuator workload. The STR and TSR configurations, which lack the central supporting joint, had to compensate by driving the platform entirely through their three legs, leading to higher peak speeds and forces in the actuators. Such requirements could translate to greater motor stress, higher energy consumption, and potentially more rapid wear or need for cooling in a physical device. In contrast, the STC and TSC designs kept peak actuator forces and velocities low. From an energy efficiency and safety standpoint, this is a crucial advantage—lower actuator effort not only implies reduced power draw (beneficial for battery-powered or mobile systems) but also smoother, safer interaction forces with the patient. Excessive speeds or forces in a rehabilitation robot can pose risks, so the ability of STC/TSC to operate more gently while still accomplishing the task is highly desirable. These results reinforce the notion that parallel robots with additional passive constraints can achieve better force economy, which in turn supports longer-term goals like portable operation and improved patient comfort.

Despite their advantages, the centrally coupled designs do exhibit a limitation in terms of mechanical range of motion. Both STC and TSC significantly restricted the translational movement of the platform’s center of gravity in our simulations—effectively, the platform was mostly rotating about a fixed point. This was evident in Figure 10, where the CG displacement for those configurations was negligible compared to STR and TSR. In contrast, the STR and TSR mechanisms, lacking the central support, allowed a much larger vertical excursion of the platform. In practical terms, the STR/TSR offer a wider workspace (greater platform travel), which could be beneficial for rehabilitation exercises requiring extensive limb movements, whereas the STC/TSC prioritize stability over range. In practical terms, a smaller platform travel might limit the versatility of exercises or the range of limb motion the robot can accommodate. For instance, certain rehabilitation routines might require a greater vertical movement or reach that the STC/TSC architectures, in their current form, cannot provide. The STR and TSR mechanisms, while less precise and more demanding on actuators, allowed a larger workspace (greater platform excursion) which could be beneficial for exercises requiring extensive limb movement. This trade-off between stability and workspace is a critical point for designers: a highly constrained mechanism offers better control and safety, whereas a less constrained mechanism offers more flexibility and range. The optimal choice may therefore depend on the specific therapeutic application—e.g., finger or wrist rehabilitation might favor precision and rigidity, whereas leg or hip therapy might require a larger range of motion. It should also be noted that our AHP decision model weighted control accuracy and force efficiency most heavily (each 30%), which influenced the final ranking. If a different weighting scheme were used—for example, prioritizing range of motion or speed more—the preferred design might change. Nonetheless, the current weighting was informed by the rehabilitation context: patient safety and precise force/motion control were deemed paramount, aligning with clinical priorities to avoid unintended aggressive movements. Within this context, the STC configuration’s top AHP score (0.80) indicates that it provides the best balance of precision, low effort, and sufficient speed/range for typical therapy needs. The TSC was the next-best option, slightly less optimal overall but still markedly better than the alternatives. Interestingly, although the TSR and STR designs have identical theoretical degrees of freedom, TSR outperformed STR in our analysis (notably in criteria C1–C3). This suggests that placing the spherical joint in the middle of the leg (TSR) is more advantageous than at the base (STR) when no central support is present—likely because the intermediate spherical joint improves the distribution of motion and forces within each leg, reducing internal stresses and improving control. This nuance underlines how even subtle differences in kinematic sequencing (S-T-R vs. T-S-R) can impact performance. In contrast, between the two centrally supported designs, STC edged out TSC overall. The STC architecture (spherical at base, universal at platform, plus central sphere) achieved slightly better accuracy and force results in the simulation than TSC (translational at base, spherical in leg, universal at platform plus central sphere). One possible explanation is that having the spherical joint at the base (as in STC) yields more unconstrained orientations for the leg to adapt to platform rotations, thereby minimizing internal stresses and control error. TSC, with the prismatic actuator at the base, might introduce a stiffer connection to the ground that marginally increases error under dynamic conditions. However, the differences between STC and TSC were not large—both are high-performing configurations—and further studies would be needed to conclusively determine why STC performs better and whether that holds under all conditions.

Overall, the discussion of results confirms the initial hypothesis that parallel mechanisms can offer distinct benefits for rehabilitation, and that the choice of joint architecture has measurable consequences on performance. By systematically analyzing these designs, we have identified a clear front-runner (STC) for implementation in a therapeutic robotic platform. Importantly, these conclusions are drawn from a simulation-based study; in practice, factors such as mechanical backlash, actuator friction, and manufacturing tolerances could influence performance. The current analysis assumes ideal joints and rigid links, which is a reasonable first approximation but will need validation. Therefore, while STC is recommended by the simulation and AHP evaluation, ongoing work should consider building a prototype or conducting hardware-in-the-loop tests to verify that these advantages carry over to a physical system. Future investigations might also explore hybrid criteria (e.g., including energy consumption explicitly, or cost and complexity of construction) to refine the decision-making process. Nonetheless, the present study provides a valuable quantitative framework for comparing parallel robotic designs and underscores the efficacy of combining simulation with multi-criteria decision analysis in robotic rehabilitation research.

## 5. Conclusions

This paper presented a comprehensive comparative study of four parallel robot architectures for rehabilitation therapy, leveraging simulation modeling and an Analytic Hierarchy Process (AHP) multi-criteria decision approach. Each candidate mechanism—STR, STC, TSR, and TSC—was evaluated under identical conditions to assess key performance indicators including motion tracking accuracy, actuator speed requirements, force demands, and achievable platform motion range. The following conclusions can be drawn from the results.

Performance differences due to kinematic architecture are significant: Even though all four mechanisms share the same theoretical degrees of freedom (3 DOF) and were able to execute the target movement, their dynamic responses varied notably. The inclusion or absence of certain joint types and auxiliary couplings affected how precisely and efficiently each robot could carry out the rehabilitation movements.

Designs with a central spherical coupling (STC, TSC) outperformed the others in precision and efficiency: The STC configuration in particular achieved the lowest tracking error (enhanced control accuracy) and required the least actuator effort (low peak forces and velocities). The TSC design showed a similarly strong performance. These two architectures benefit from the extra passive spherical joint that stabilizes the platform, confirming the value of added constraints for improving control outcomes in parallel robots.

Designs without the central coupling offer greater motion range but at a cost: The STR and TSR mechanisms allowed a larger vertical displacement of the platform—an advantage in terms of workspace and potential exercise range. However, this came with trade-offs: STR and TSR exhibited higher peak forces, higher speeds, and slightly larger tracking errors, which could stress actuators and reduce control fidelity. Thus, while they may be suitable for applications needing a wide range of motion, they are less ideal when precise, gentle force delivery is the priority.

Multi-criteria ranking identified STC as the best overall solution for rehabilitation purposes: By synthesizing the simulation data through an AHP weighting scheme (emphasizing accuracy and force efficiency), the insights gained, such as the superior control accuracy of the STC configuration versus the greater workspace of the TSR, can directly inform the design of next-generation rehabilitation devices depending on clinical priorities (precision vs. range of motion). TSC was the second-highest ranked, followed by TSR and then STR. The ranking validates the intuitive expectation that a well-constrained, stable mechanism (with slight workspace sacrifice) is optimal for controlled therapeutic movements. It also quantitatively justifies the design decision, providing a clear rationale grounded in measurable performance metrics.

Implications for rehabilitation robot design: The findings underline that parallel robotic platforms can be finely tuned to meet rehabilitation needs by appropriate joint selection and topology. For instance, if a particular therapy demands extremely precise motion or low interaction forces (e.g., delicate post-stroke exercises), a configuration like STC is highly advantageous. On the other hand, if a therapy values a large range of motion (e.g., stepping or gait-like exercises) and can tolerate slightly less precision, a configuration like TSR might be acceptable. The use of AHP in this context offers a flexible tool to reprioritize criteria depending on clinical requirements, ensuring that the chosen design aligns with therapeutic goals.

In conclusion, this research demonstrates the power of integrating dynamic simulation with structured decision-making to guide the design of rehabilitation robots. Parallel mechanisms show great promise for rehabilitation due to their inherent stiffness, accuracy, and compactness, and our comparative approach helps identify which specific parallel architecture best balances the competing demands of a rehabilitation scenario.

The primary advantage of the proposed mechanisms is their ability to facilitate rehabilitation through progressively applied resistance (force) exercises, rather than relying solely on passive or unweighted movement. For example, a patient might be able to move their injured leg freely while lying in bed, but experience significant difficulty when attempting to stand and bear weight on it. This illustrates that effective limb rehabilitation must include gradually increasing muscle loading (weight-bearing exercises) in addition to kinematic exercises (simple range-of-motion movements). By enabling controlled, incremental loading of the muscles, the proposed mechanisms bridge the gap between basic movement and full weight-bearing functionality. In other words, recovery is not just about kinematics (movement alone); it also requires building muscle strength and endurance. The mechanisms we propose provide a safe and structured way to introduce this progressive muscular load, thereby promoting more comprehensive recovery and improving the patient’s ability to return to weight-bearing activities with confidence.

The recommended design (STC) will be the focus of future work, which will involve prototyping and experimental validation of its performance with human subjects. This step will bridge the simulation results to real-world practice, ensuring that our proposed optimal solution is feasible and beneficial in a clinical environment. Additionally, the methodology established here, combining physics-based simulations of robot–human interactions with AHP evaluations, can be generalized to other robotic design problems where multiple performance objectives must be harmonized. Ultimately, ensuring that robotic rehabilitation devices are optimally designed for both patient safety and therapeutic effectiveness will accelerate their adoption and impact in clinical practice, ensuring that rehabilitation robots are optimally designed for safety and therapeutic efficacy will expedite their adoption in clinical settings, ultimately improving patient outcomes. Our findings provide designers with a clear rationale for selecting parallel robot architectures based on therapeutic priorities. For instance, if precise motion control and gentle force delivery are paramount, an STC configuration is recommended; if a wide range of motion is needed and slight precision trade-offs are acceptable, a TSR configuration might be suitable. By quantitatively identifying the optimal mechanism (STC) for rehabilitation, this research offers practical guidance for future device development, which we intend to pursue through prototyping and clinical testing.

## Figures and Tables

**Figure A6:**
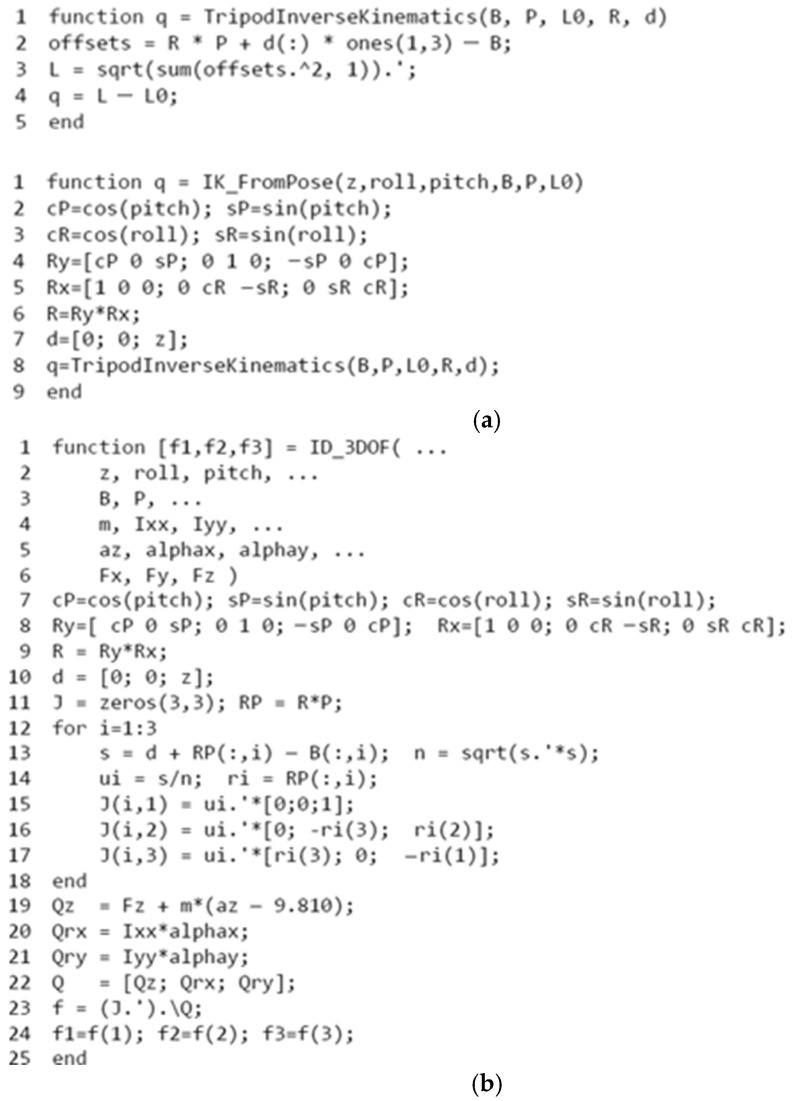
Algorithms in Matlab. (**a**) Inverse Kinematics; (**b**) Inverse Dynamics.

## Data Availability

The raw data supporting the conclusions of this article will be made available by the authors on request.
